# Red blood cell transfusion associated with increased morbidity and mortality in patients undergoing elective open abdominal aortic aneurysm repair

**DOI:** 10.1371/journal.pone.0219263

**Published:** 2019-07-11

**Authors:** Charlotte Wedel, Cecilie M. Møller, Jacob Budtz-Lilly, Nikolaj Eldrup

**Affiliations:** 1 Department of Cardio-Thoracic and Vascular Surgery, Aarhus University Hospital, Aarhus, Denmark; 2 Danish Vascular Registry, Aarhus University Hospital, Aarhus, Denmark; China Medical University Hospital, TAIWAN

## Abstract

**Background:**

Red blood cell (RBC) transfusions are associated with increased mortality and morbidity. The aim of this analysis was to examine the association between RBC transfusions and long-term survival for patients undergoing elective open infrarenal abdominal aortic aneurysm (AAA) repair with up to 15 years of follow-up.

**Methods:**

Prospective cohort study using data from The Danish Vascular Registry from 2000–2015. Primary endpoint was all-cause mortality. Secondary endpoints were in-hospital complications. Transfused patients were divided into subgroups based on received RBC transfusions (1, 2–3, 4–5 or > 5). Using Cox regression multi-adjusted analysis, non-transfused patients were compared to transfused patients (1, 2–3, 4–5, >5 transfusions) for both primary and secondary endpoints.

**Results:**

There were 3 876 patients included with a mean survival of 9.1 years. There were 801 patients who did not receive transfusions. Overall 30-day mortality was 3.1% (121 patients) and 3.6% (112) for all transfused patients. For the five subgroups 30-day mortality was: No transfusions 1.1% (9 patients), 1 RBC 1.2% (4 patients), 2–3 RBC 2.2% (26 patients), 4–5 RBC 1.9% (14 patients) and > 5 RBC 7.9% (68 patients). After receiving RBCs, the hazard ratio for death was 1.54 (95% CI 1.27–1.85) compared to non-transfused patients. There was a significant increase in mortality when receiving 2–3 RBC: HR 1.32 (95% CI 1.07–1.62), 4–5 RBC: 1.64 (1.32–2.03) and >5 RBC: 1.96 (1.27–1.85) in a multi-adjusted model.

**Conclusion:**

There is a dose-dependent association between RBC transfusions received during elective AAA repair and an increase in short- and long-term mortality. Approximately 25% of included patients had preoperative anemia. These findings should raise awareness regarding potentially unnecessary and harmful RBC transfusions.

## Introduction

Anemia is a well-known risk factor for patients undergoing non-cardiac surgery.[1–3] In the clinical setting, the most common indications for red blood cell (RBC) transfusions are anemia, acute blood loss and risk of inadequate oxygen supply to the tissue.[4–10] Studies have shown great variation in the amount of RBC transfusions used for similar procedures across hospitals and countries.[9, 11–13] This suggests that consensus, or adherence thereto, regarding optimal transfusion levels varies, and this appears to be true for vascular surgery patients as well.[11, 12] For patients with an abdominal aortic aneurysm (AAA), the conventional practice in Denmark is to offer RBC transfusions when the hemoglobin level falls below 9.7 g/dL (6.0 mmol/L), based in part on the high burden of atherosclerosis in these patients.[14] Recent Cochrane reviews comparing patients from different clinical specialties found no increase in mortality or overall morbidity when utilizing a restrictive transfusion strategy.[15, 16] Choosing a restrictive transfusion threshold of 7 g/dL to 8 g/dL, did, however, reduce the proportion of patients exposed to RBC transfusion by 43% with no impact on mortality or morbidity.[16]

Evidence regarding specifically vascular surgery patients is somewhat limited, although two studies have suggested an association between perioperative RBC transfusion and higher 30-day mortality and morbidity.[11, 17] In addition, RBC transfusion in conjunction with lower extremity bypass surgery is known to be a risk factor for postoperative wound infections.[18]

We studied the impact of RBC transfusions on long-term survival of patients undergoing elective open repair for abdominal aortic aneurysm. Furthermore, we studied whether there was a dose-dependent association between the received dosage of RBC transfusions and mortality, as well as in-hospital complications.

## Methods

### Study population

The study included prospectively collected data on patients undergoing elective open abdominal aortic aneurysm repair from January 1, 2000 to December 31, 2014. All patients were treated at one of 10 vascular surgery departments in Denmark. All patients who underwent endovascular aneurysm repair (EVAR) were excluded. All patients with juxtarenal, iliaca, symptomatic, ruptured, mycotic, dissecting, pseudo or thrombosed aneurysms were also excluded. Data were collected from the Danish Vascular Registry (Karbase), a Danish nationwide database, in which all vascular procedures are registered. The registry has previously been validated[19]. Patient survival status was determined on 1 July 2015 from the Danish National Population Registry (civil personal registry (CPR)). Retrieval of data was approved by The Danish Data Protection Agency (Datatilsynet), record number 2015-41-4135. Danish registry data are available to researchers and use of this data does not require informed consent as per Danish law. All personally identifiable data was fully anonymized. Patients were excluded if there were no endpoint data, e.g. foreign patients with no Danish civil personal registry code, or no data on perioperative transfusions. Perioperative transfusion was defined as any RBC transfusions given during the hospital admission.

### Endpoints

The primary endpoint was all-cause mortality. Association was first investigated between any transfusion and mortality, thereafter for a dose dependent association and lastly for an association if initial mortality was censored.

Secondary endpoints included in-hospital complications, defined as: dialysis, intestinal ischemia, pulmonary complications, cardiac complications, embolus and acute tubulointerstitial nephritis, each of which were analyzed individually. For detailed definitions of in-hospital complications, see S1A Appendix.

### Study variables

Variables included age, gender, body mass index (BMI) (kg/m^2^), as well as preoperative values of hemoglobin and serum creatinine. Anemia was defined as hemoglobin <13.4 g/dL (8.3 mmol/L) for men and <11.8 g/dL (7.3 mmol/L) for women. Data regarding smoking as well as documented cardiac or cerebrovascular disease, diabetes mellitus, hypertension, and pulmonary disease were also collected. For detailed definitions, see S2A Appendix. Missing continuous values were given the median value, while missing categorical values were assigned their own group. Numbers of missing values on preoperative comorbidities are listed in S2B Appendix.

### Statistical analysis

The association between RBC transfusion and long-term mortality was examined with two models:

- No transfusion (reference group) vs. transfusion
- Dose dependence; No transfusion (reference group) vs. 1 unit, 2–3 units, 4–5 units and > 5 units of RBC

A Cox proportional hazards regression hazard ratio (HR) for death according to transfusions (no/yes) and number of transfusions (subgroups: 0, 1, 2–3, 4–5 or > 5 transfusions) were calculated along with 95% confidence interval (CI). This was done adjusting for age and gender, as well as a multi-variable analysis (adjusting for age, gender, preoperative hemoglobin and creatinine, bleeding, BMI, smoking, diabetes, hypertension, cerebrovascular, cardiac and respiratory disease). Differences in baseline characteristics between the transfusion group and the non-transfusion group were analyzed with the *X*^*2*^*-*test for categorical variables and the Kruskal-Wallis test with Bonferroni correction for continuous variables.

Survival was estimated using Kaplan-Meier curves with log-rank tests for statistical comparisons.

The χ^2^ -test was applied to compare categorical preoperative variables and postoperative complications between the non-transfused group and the four transfused subgroups or combined transfused group.

In order to determine an association between RBCs and secondary in-hospital complications, two logistic regression models were constructed; Model I: adjusted for age and gender, Model II: adjusted for age, gender, bleeding, preoperative hemoglobin, preoperative creatinine, and preoperative morbidity (cardiac disease, pulmonary disease, diabetes, previous cerebrovascular events and hypertension).

To evaluate a potential change after the introduction of leukocyte-depleted blood, an analysis was also carried out regarding changes in the 30-day mortality during the 15-year follow-up for the combined patient group and for patients receiving RBCs (S3A Appendix).

To investigate if postoperative transfusion was causing the impact on reduced survival, we investigated survival with Kaplan-Meier analysis depending on time for transfusion, only intraoperatively versus intraoperatively and postoperatively, and stratified for number of transfusions (S4 Appendix).

A p-value <0.05 was considered statistically significant. Continuous values are given as mean values with standard deviation (±SD) in parenthesis.

## Results

**Fig 1:**
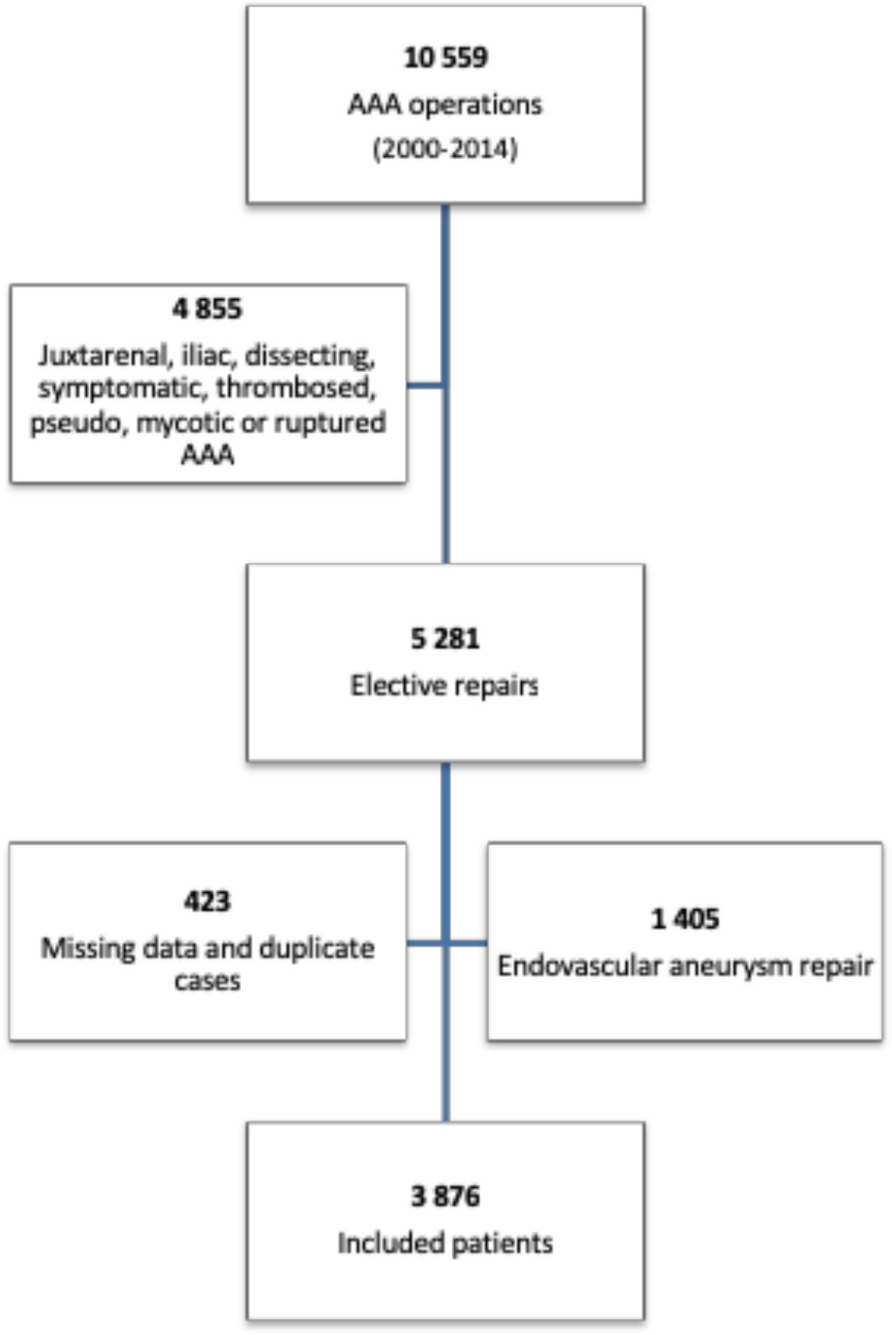
Flow chart of patient selection process with excluded and included patients. Data were retrieved from The Danish Vascular Registry during the period of January 1, 2000 to December 31, 2014. AAA: abdominal aortic aneurysm.

A total of 10 559 AAA operations were registered in The Danish Vascular Registry. Only elective, asymptomatic patients with infrarenal AAA were included, though not including endovascular repairs. A total of 3 876 patients were included in the study (Fig 1). 18.5% (717) were women and 81.5% (3 159) men. 54% (2 089) had aortic repair with a tube graft and 46% (1 787) with a bifurcated graft. The mean hemoglobin for all patients upon admission was 13.8 (SD ±1.5) g/dL (8.6 ±0.9 SD mmol/L). The mean hemoglobin in g/dL (±SD) for the five groups was as follows: No transfusion: 14.2 (±1.5), 1 RBC 13.9 (±1.5), 2–3 RBC 13.9 (±1.5), 4–5 RBC 13.5 (± 1.5) and > 5 RBC 13.4 (± 1.6). A total of 25.4% (986) had preoperative anemia, 11.9% (85) of women and 28.5% (901) of men. Further baseline characteristics are given in Table 1. During the 15-year follow-up period, 38% (1 465) patients died, with a mean survival of 9.1 years (95% CI 8.9–9.3).

**Table 1:** Baseline characteristics, preoperative variables, and perioperative bleeding.

	**0** **N = 801**	**1** **N = 329**	**2–3** **N = 1 165**	**4–5** **N = 724**	**>5** **N = 857**	**All patients** **N = 3 876**
**Gender**						
% (n) of male patients	83.0 (665)	79.6 (262)	81.2 (946)	80.2 (581)	82.3 (705)	81.5 (3 159)
% (n) of female patients	17.0 (136)	20.4 (67)	18.8 (219)	19.8 (143)	17.7 (152)	18.5 (717)
**Age (years), mean (±SD)**	68.9 (±7.2)	69.8 (±7.1)	70.4 (±6.7) *	70.8 (±6.9) *	71.2 (±7.1) *	70.3 (±7.0)
**Hemoglobin (g/dL), mean (±SD)**	14.17 (±1.5)	13.9 (±1.5)*	13.9 (±1.5) *	13.5 (±1.5) *	13.4 (±1.6) *	13.8 (±1.5)
% (n) **of anemic patients**	15.6 (125)	26.1 (86)	23.8 (277)	29.3 (212)	33.4 (286)	25.4 (986)
% (n) of anemic male patients	17.9 (119)	29.8 (78)	27.6 (261)	25.7 (186)	36.5 (257)	28.5 (901)
% (n) of anemic female patients	4.4 (6)	3.1 (8)	7.3 (16)	18.2 (26)	19.1 (29)	11.9 (85)
**Creatinine (mmol/L), mean (±SD)**	92.1 (±42.7)	91.1 (±34.0)	97.4 (±58.4)	98.9 (±53.1) *	105.4 (±70.1) *	97.8 (±56.0)
**BMI (kg/m^2^), mean (±SD)**	26.5 (±4.2)	26.2(±4.2)	26.1 (±3.4)	26.5 (±4.4)	26.4 (±4.2)	26.3 (±4.2)
**Smoker**	85.5 (685)	85.2 (280)	82.4 (960)	82.6 (598)	82.0 (703)	83.2 (3 226)
**Diabetes**	7.4 (59)	9.4 (31)	8.2 (95)	9.8 (71)	10.7 (92)	8.9 (348)
**Cerebrovascular disease**	8.3 (67)	7.3 (24)	9.9 (115)	8.4 (61)	11.4 (97)	9.4 (364)
**Hypertension**	64.2 (514)	59.0 (194)	59.9 (697)	64.7 (468)	62.8 (538)	63.2 (2411)
**Cardiac disease**	30.1 (241)	31.6 (104)	32.1 (374)	33.1 (240)	37.9 (325) *	33.1 (1284)
**Respiratory disease**	11.4 (91)	15.8 (52) *	13.7 (159)	14.8 (107) *	17.4 (149) *	14.4 (558)
**Perioperative bleeding (mL), mean (±SD)**	1 026 (±545)	1 143 (±643)	1 627 (±765) *	2 178 (±952) *	3 379(±2 682) *	1 952 (±1 660)

Categorical data are % (n). Differences was compared to the group with no transfusion.

*P<0.05 after correction for multiple testing.

### Blood loss and transfusions

**Fig 2:**
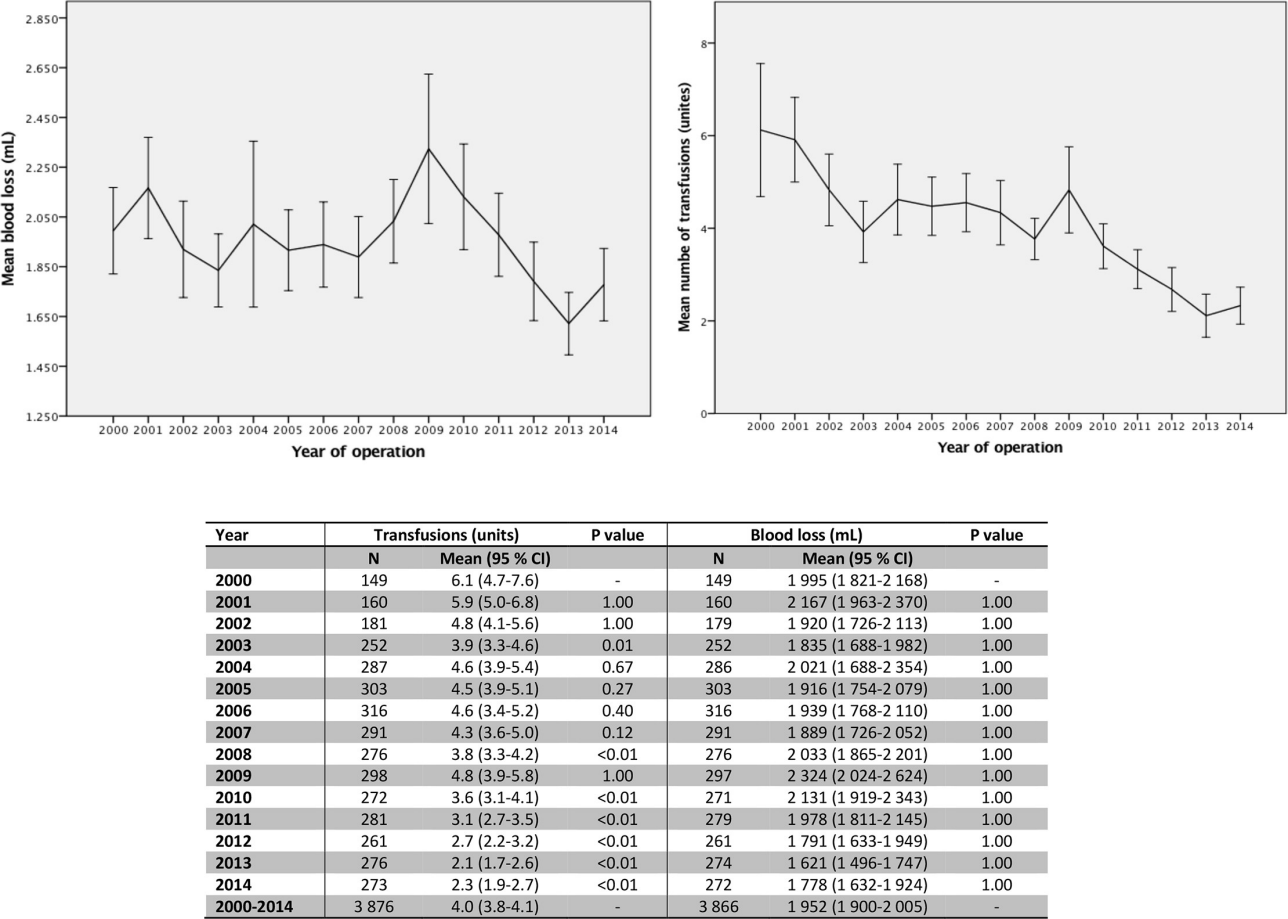
Development in transfusions and blood loss during 15-year follow-up period. Upper left: Mean blood loss (mL) per patient across time. Upper right Mean number of transfusions per patient across time. Lower: Development in transfusions and blood loss from January 1, 2000 to December 31, 2014. P value calculated with year 2000 as reference.

The mean perioperative blood loss was 1 952 mL. A total of 3 075 patients received at least one unit of RBC during hospital stay, while 801 patients received no transfusions. The trend in blood loss and transfusions during the study period is displayed in Fig 2. The use of RBC transfusions declined from 6.1 (95% CI 4.7–7.6) units/patient in 2000 to 2.3 (1.9–2.7) in 2014.

### Survival

**Fig 3:**
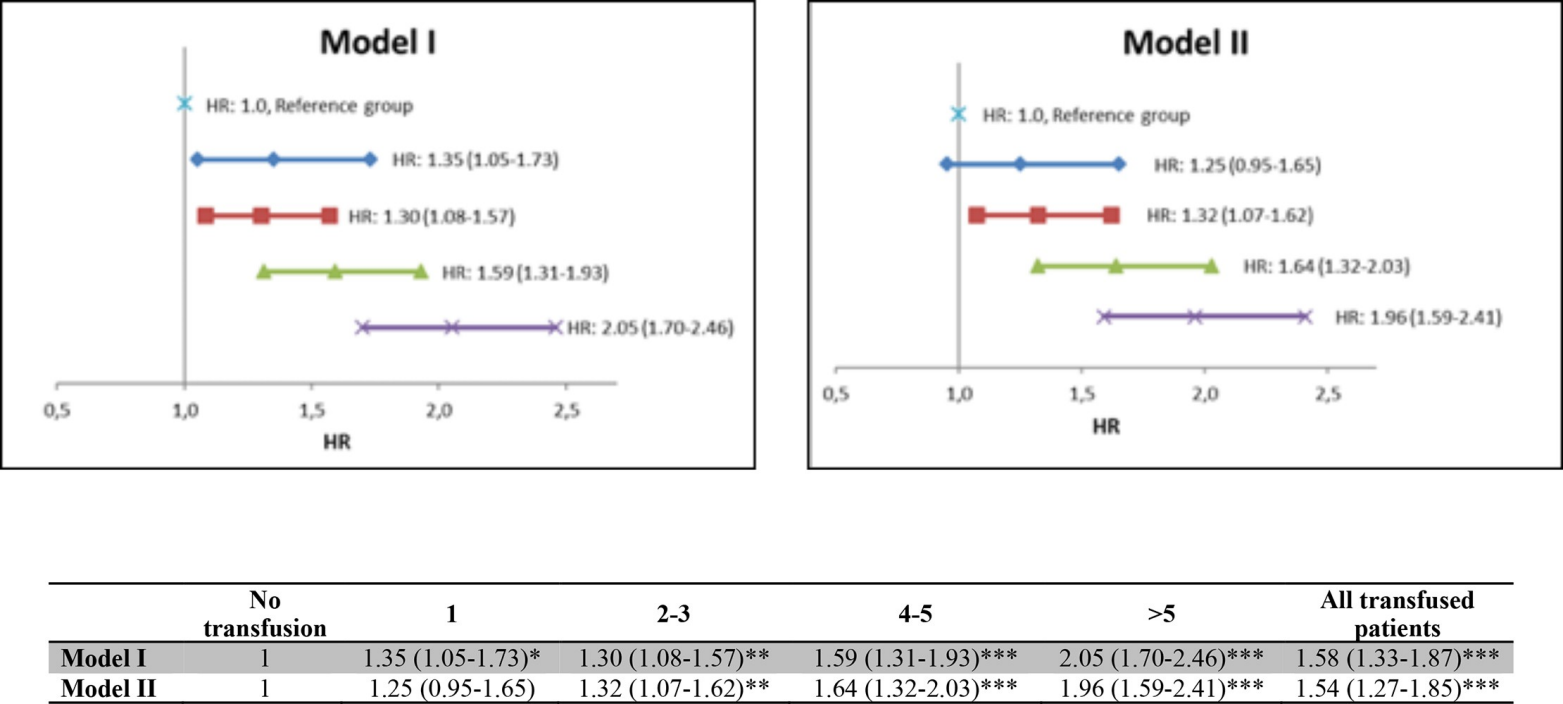
Hazard ratio (95% CI) for mortality, all transfused patients and dose-dependent subgroups compared to non-transfused patients (reference group). Above: Forest plot based on HR in table below Forest plot. Model I: Adjusted for age and gender. Model II: Adjusted for all baseline variables and characteristics (gender, age, preoperative hemoglobin and creatinine, bleeding, BMI, smoking, diabetes, hypertension, cerebrovascular, cardiac and respiratory disease). * P value < 0.05. ** P value < 0.01. *** P value < 0.001. The Kaplan-Meier plot shows a significant difference in survival between the non-transfused vs. transfused group, log rank p < 0.00001 (Fig 4).

After adjusting for gender, age, preoperative hemoglobin and creatinine, bleeding, BMI, smoking, diabetes, hypertension, cerebrovascular, cardiac and respiratory disease, the HR for all-cause mortality for all transfused patients, when compared to non-transfused, was 1.54 (95% CI 1.27–1.85)) (Fig 3, Model II).

**Fig 4:**
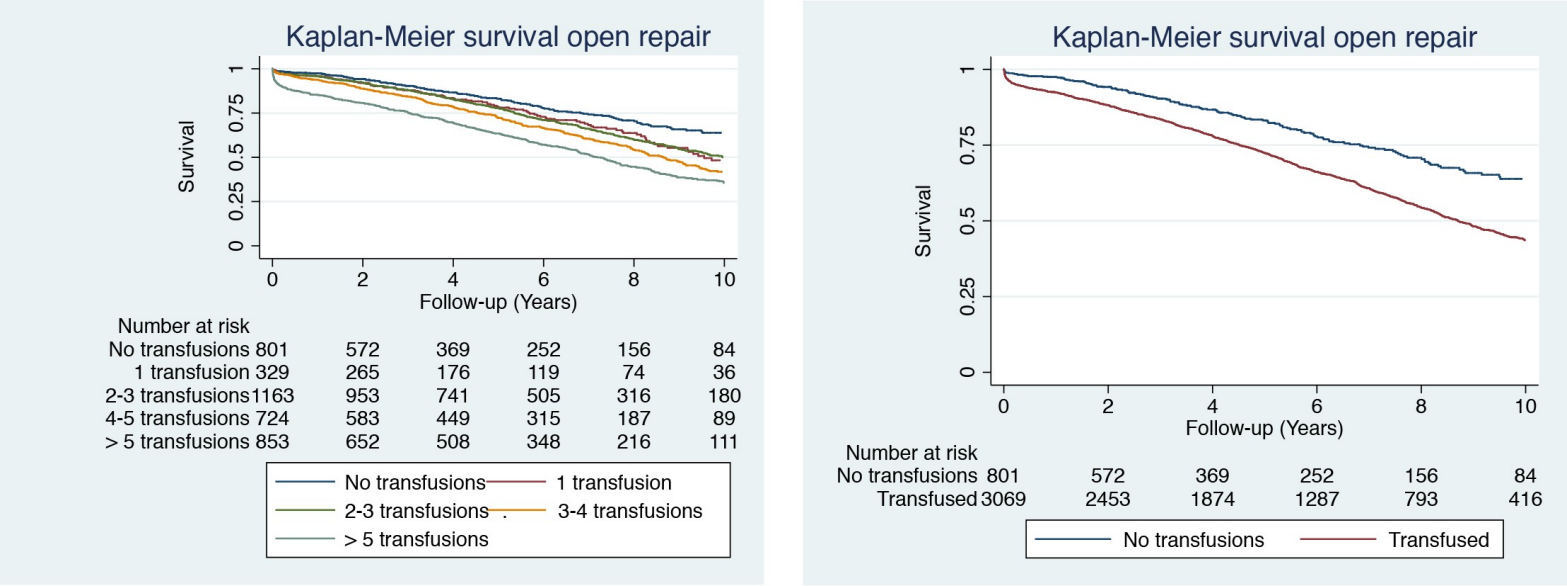
Kaplan-Meier survival curves for patients undergoing open repair. Data are stratified by number of received transfusions (0, 1–2, 2–3, 4–5 or >5) (left) or by transfused or non-transfused patients (right). Follow-up is shown for up to 10 years after aortic repair.

The 30-day mortality for all included patients was 3.1% (121 patients) and 3.6% (112) for all transfused patients. The 30-day mortality for the five groups was as follows: no transfusions 1.1% (9 patients), 1 RBC 1.2% (4 patients), 2–3 RBC 2.2% (26 patients), 4–5 RBC 1.9% (14 patients) and > 5 RBC 7.9% (68 patients) (S5 Appendix).

For the five subgroups, mean survival after repair was: No transfusion 9.8 years (95% CI 9.4–10.1), 1 RBC 9.2 years (8.5–9.9), 2–3 RBC 9.6 years (9.2–9.9), 4–5 RBC 8.7 years (8.3–9.2) and > 5 RBC 7.6 years (7.2–8.0) respectively (Table 2).

**Table 2:** Survival after aortic repair.

**Duration of follow-up**	**No transfusions** **N = 801**^1^	**1** **N = 329**^1^	**2–3** **N = 1 165**^1^	**4–5** **N = 724**^1^	**>5** **N = 857**^1^
**30 days**	99 (792)	99 (4)	98 (26)	98 (14)	92 (68)
**1 year**	97 (742)	96 (305)	96 (1087)	94 (662)	85 (719)
**2 years**	94 (627)	92 (274)	92 (988)	89 (597)	80 (662)
**3 years**	90 (502)	88 (235)	88 (884)	84 (538)	75 (598)
**4 years**	87 (402)	83 (191)	82 (775)	78 (468)	69 (525)
**5 years**	83 (329)	78 (158)	78 (664)	72 (398)	63 (449)
**6 years**	78 (268)	73 (130)	71 (541)	66 (333)	57 (370)
**7 years**	74 (221)	68 (106)	66 (445)	60 (266)	51 (300)
**8 years**	71 (174)	64 (84)	60 (349)	54 (204)	44 (232)
**9 years**	66 (129)	55 (57)	55 (262)	48 (151)	38 (173)
**10 years**	64 (97)	48 (40)	50 (198)	42 (105)	35 (128)
**Mean survival**, years (95%CI)	9.8 (9.4–10.1)	9.2 (8.5–9.9)	9.6 (9.2–9.9)	8.7 (8.3–9.2)	7.6 (7.2–8.0)

Survival 30 days and up to 10 years after aortic repair, % (n). Mean survival in years (95% CI).

^1^ Number of participants at start of follow-up.

After adjusting for gender, age, BMI, preoperative hemoglobin, creatinine, smoking, diabetes, hypertension, bleeding, cerebral, cardiac and pulmonary disease, the hazard ratio (HR) for mortality was significantly higher for the three groups receiving > 1 unit of RBC: 2–3 RBC: HR 1.32 (95% CI, 1.07–1.62), 4–5 RBC: 1.64 (1.32–2.03), > 5 RBC: 1.96 (1.59–2.41) (Fig 3, Model II) when compared to the non-transfused patients.

As a proxy variable for the extension of disease and complexity of the procedure, we investigated whether the type of graft, i.e. tube versus bifurcated graft, affected the multi-adjusted Model II. The difference was statistically non-significant, p = 0.492. Also, we investigated if operative time as a continuous variable could be a proxy-variable for complicated surgery. This also did not render a significant contribution to model II, p = 0.489. Neither proxy variables were therefore included in the model.

### Long-term impact of blood transfusion

After excluding the first 30 days, as well as one and five years following repair, the Kaplan-Meier survival analysis show a significant difference in survival for patients not receiving RBC transfusions compared to those patients who received RBCs, (log-rank test overall comparison p < 0.001, p < 0.001 and p < 0.001, for each comparison) (S3C Appendix). Furthermore, the Cox hazard ratio multi-adjusted analysis (S3 Appendix, Model II), including only patients surviving more than 30 days, revealed a HR of 1.43 (95% CI 1.15–1.78) for patients receiving 4–5 transfusions versus none and a HR of 1.42 (1.13–1.77) for those receiving >5 versus none. Similarly, survivors of more than one year receiving 4–5 transfusions versus none showed a HR of 1.37 (1.09–1.71), while survivors receiving >5 versus none, HR 1.36 (1.07–1.74). For survivors of more than five years, there was no longer any difference in hazard ratios. That is, survivors receiving 4–5 transfusions versus none, HR 1.37 (0.98–1.90), and survivors receiving >5 versus none, HR 1.33 (0.94–1.89).

### Timing of transfusion

Kaplan-Meier survival curves shows that receiving transfusion postoperatively compared to only intraoperatively was associated to reduced survival (S4A Appendix), log Rank (Mantel-Cox P< 0.0001). The dose-dependent association is preserved for both patients receiving only transfusion intraoperatively (S4B Appendix) as well as for patients receiving transfusion both intra- and postoperatively (S4C Appendix), log Rank (Mantel-Cox P< 0.0001).

### Secondary outcomes

The Cox hazard ratio for postoperative in-hospital complications showed an increasing risk for patients receiving RBCs when compared to non-transfused patients. This was moreover true after adjusting for gender, age, bleeding, preoperative hemoglobin, preoperative creatinine, diabetes, hypertension, cerebrovascular, cardiac and pulmonary disease (Table 3, Model II and S6 Appendix).

**Table 3:** Dose-dependent hazard ratio (95% CI) for selected postoperative in-hospital complications.

	**Model I**					
		**No** **transfusions**	**1**	**2–3**	**4–5**	**> 5**
**Dialysis**		1	3.65 (0.61–21.94)	2.72 (0.57–12.84)	7.16 (1.61–31.87)*	37.56 (9.18–153.70)***
**Intestinal ischemia**		1	1.58 (0.26–9.52)	2.91 (0.83–10.24)	5.38 (1.55–18.67)**	20.30 (6.34–65.04)***
**Pulmonary complications**		1	1.72 (1.04–2.85)*	1.43 (0.97–2.10)	1.76 (1.17–2.65)**	3.49 (2.42–5.03)***
**Cardiac complications**		1	0.98 (0.50–1.90)	1.59 (1.03–2.45)*	1.45 (0.90–2.33)	3.90 (2.59–5.86)***
**Embolus**		1	3.60 (1.13–11.42)*	2.65 (0.98–7.19)	1.91 (0.62–5.89)	5.01 (1.89–13.24)**
**Acute tubulointerstitial nephritis**		1	3.18 (1.09–9.24)*	1.74 (0.68–4.47)	4.38 (1.78–10.75)**	13.92 (6.04–32.08)***
	**Model II**					
		**No** **transfusions**	**1**	**2–3**	**4–5**	**> 5**
**Dialysis**		1	6.77 (0.70–65.74)	3.72 (0.44–31.12)	10.05 (1.27–79.34)*	54.09 (7.37–397.05)***
**Intestinal ischemia**		1	2.58 (0.34–19.63)	4.45 (0.91–21.80)	10.08 (2.09–48.56)**	34.30 (7.37–159.57)***
**Pulmonary complications**		1	1.75 (1.03–2.98)*	1.43 (0.94–2.19)	1.89 (1.20–2.99)**	3.83 (2.43–6.05)***
**Cardiac complications**		1	0.85 (0.42–1.70)	1.52 (0.97–2.40)	1.37 (0.82–2.28)	3.64 (2.26–5.87)***
**Embolus**		1	4.73 (1.36–16.47)*	2.85 (0.94–8.68)	2.16 (0.61–7.60)	4.95 (1.56–15.66)**
**Acute tubulointerstitial nephritis**		1	4.02 (1.26–12.82)*	2.04 (0.72–5.81)	4.79 (1.74–13.20)**	13.72 (5.23–35.98)***

**Model I:** adjusted for gender and age.

**Model II:** adjusted for gender, age, bleeding, preoperative hemoglobin, preoperative creatinine, cardiac disease, pulmonary disease, diabetes, previous cerebrovascular events and hypertension.

* P value < 0.05

** P value < 0.01

*** P value < 0.001

## Discussion

In this prospective cohort study, transfusion of four or more units of RBCs was associated with an increase in mortality. The increase in mortality was maintained throughout the 15 years of follow-up. Moreover, the increased risk in mortality and morbidity was found to be dose-dependent. A similar association has previously been demonstrated in a recent study investigating the effect of RBC transfusions in conjunction with lower extremity bypass surgery.[18] Our results are also in concordance with existing studies on patients undergoing cardiac surgery, showing an association between RBC transfusions and an increase in mortality and postoperative infections.[20–24] As the Kaplan-Meier curves show, there is still an increasing difference in mortality between the groups as time passes after aortic repair, indicating a longer lasting response after receiving RBCs. To our knowledge, no studies have investigated the dose-dependent relationship between RBC transfusions and mortality and morbidity after more than five years in patients undergoing vascular surgery.

The discussion regarding optimal transfusion triggers has resulted in more restrictive transfusion policy in many countries. Clinical analyses are difficult to perform, just as retrospective evaluations are limited by considerable confounders (see Limitations below). Isolated studies of vascular surgery patients are sparse. Decisions as to when or whether to administer RBC transfusion are fraught with individual bias and opinions, no doubt influenced by heterogeneous patient comorbidities, surgical team preferences, and input from other specialists, e.g., anesthesiologists.

Recent reviews have concluded that restrictive transfusion strategies are safe in most clinical settings, and liberal transfusion strategies could be associated with possible harm.[15, 16] Interestingly, a dose-dependent association, as observed above, would unlikely be uncovered in larger types of meta-analyses. The results presented above, in conjunction with the mentioned reviews, a more restrictive approach should be considered regarding RBC transfusions for patients undergoing elective open AAA repair. In the very least, this should highlight the need for randomized clinical trials to better ascertain the optimal trigger point for administrating transfusions and perhaps even the optimal postoperative hemoglobin level. A restrictive transfusion trigger of 7–8 g/dL could possibly be used without fear of adverse risks.[16]

To obtain a uniform patient cohort, we focused only on elective, infrarenal open aortic aneurysm repairs. All patients were evaluated preoperatively in order to determine eligibility for open surgery, thus partially explaining the similar baseline characteristics between patient groups. Even after adjusting for several preoperative variables, our results still show a consistent significant difference in mortality between patients who received transfusions and those who did not. During the observation period, treatment and secondary medical prophylaxes have improved. This has led to increased survival and could potentially impact the long-term results. However, this impact should presumably impact all patient groups equally.

Studies have investigated transfusion-associated immunomodulation,[25–28] but little is still known about this response. Transfusion-associated immunomodulation is associated with transfusion of non-leukocyte depleted blood. During our follow-up time there has been a change in blood transfusion practices after the introduction of leukocyte depleted blood. In Denmark, the use of leukocyte-depleted blood has increased from 12% in 2000 to 100% in 2011. Although the use of non-leukocyte depleted blood could explain some of our results, the 30-day mortality for patients receiving RBCs was unchanged during our 15-year follow-up period (S3A Appendix). Our findings support previous findings that further investigation into the mechanisms of these effects is warranted.[29]

The temporal trend in blood transfusions during the study period reveals a decline in the use of RBCs from approximately 345 000/year in 2002 to 295 000/year in 2011. This decline partly reflects a more restrictive blood transfusion policy following the introduction of the national guidelines on RBC transfusions from The Danish Health and Medicines Authority in 2007.[14] In addition, the ongoing introduction of several minimally invasive procedures, like cell-salvage or anti-fibrinolytic drugs, also results in fewer RBC transfusions as a result of less bleeding.[30, 31] Our study cohort was comprised only of elective repairs and, therefore, any anticoagulant medicine would have been paused prior to AAA repair. Interestingly, during follow-up, although there was significant decline in the amount of blood transfusions, there was no corresponding drop in mean blood loss (Fig 2).

### Limitations

Uninvestigated baseline characteristics of patients receiving RBC transfusions may have been different from patients not receiving transfusions. Multi-adjusted Cox regression analysis was used to test the effect of potential confounders and to adjust for differences. This adjustment should dampen the potential effect of bias seen for transfusion levels in patients with and without cardiac and pulmonary disease.

A small group of patients had major perioperative bleeding and larger RBC transfusions, suggestive of complications, which then becomes difficult to quantify and differentiate in their potential impact on mortality. A sensitivity analysis was therefore performed by eliminating this group of patients, and the dose-dependent relationship is still apparent. Moreover, we attempted to evaluate variables that may reflect technically complex or complicated procedures, such as operative time and choice of graft. Incorporation of these variables did not alter the results from the Cox regression model.

A weakness of the present study is the missing information on indication for transfusion postoperatively, whether they were given on hemoglobin level or due to symptoms.

The strengths in this study are the large unselected national cohort, the validity of the Danish Vascular Registry,[19] and the long follow-up of up to 15 years by national registries. Due to this national inclusion, local differences in indication due to symptoms and level of hemoglobin, will only marginally affect the general transfusion rate observed in the present study.

Several factors have changed during this time, i.e. the use of leukocyte-depleted blood, surgical techniques, hemostatic drugs, cell savers, and other clinical practices, which are difficult to account for in this study design.

Unfortunately, only preoperative hemoglobin values were available for the analysis. There is no information about the nadir hemoglobin levels nor the values for which the decision was made to transfuse the patient. Even though the hemoglobin was not significantly different between our patient groups, it was remarkable that all groups had mean hemoglobin levels below normal. In elective patients, prophylactic measures could be taken to minimize or avoid this anemia. With higher preoperative hemoglobin, more bleeding would be tolerated before reaching transfusion threshold or symptoms of anemia and thereby potentially reducing the necessary amounts of RBCs. Our results show significant dose-dependent associations. The transfused patients never caught up with the lower mortality of the non-transfused patients. We believe that any reduction in number of transfusions could improve the patients long-term outcome.

Clearly, more evidence is needed for transfusion limits among vascular patients. Prospective randomized controlled trials investigating transfusion limits both perioperatively and postoperatively should be urged, and ideal follow-up should be planned for a minimum of five years so late responses can be investigated.[15]

## Conclusion

Patients undergoing elective infrarenal AAA repair have an increased long-term and short-term mortality and morbidity following multiple transfusions. The association with mortality and morbidity is dose-dependent. Furthermore, there is an increasing risk of postoperative morbidity following RBC transfusion. Patients receiving RBCs do not appear to lose this early increased mortality, even 15 years following aortic repair. These findings should increase the awareness of preoperative anemia and serve as a caution against possible unnecessary transfusions.

## Supporting information

S1 Appendix **a:** Definition of postoperative in-hospital complications.
**b:** Surgical postoperative in-hospital complications. Data are summarized by % (n).
(DOCX)

S2 Appendix**a:** Definition of preoperative comorbidities.The variables were divided in a “Yes” or “No” group as depicted above.Body mass index (BMI) was defined as: BMI = $\frac{Weight\;(Kg)}{{\left(Height(m)\right)}^{2}}$**b:** Missing data on preoperative comorbidities % (N).(DOCX)

S3 Appendix **a:** Temporal trend in 30-day mortality from January 2000 to December 2014, % (N). During 2000 and 2001 all included patients received a minimum of 1 RBC transfusion.
**b:** Mortality Hazard ratio (95% CI) for dose-dependent subgroups compared to non-transfused patients after excluding patients who did not survive the first 30 days, one year or five years.
**Model I:** Mortality hazard ratio adjusted for age and gender.
**Model II:** Mortality hazard ratio adjusted for all baseline variables and characteristics (gender, age, preoperative hemoglobin and creatinine, bleeding, BMI, smoking, diabetes, hypertension, cerebrovascular, cardiac and respiratory disease).
**c:** Dose-dependent survival for all patients included in the study after excluding the first 30 days, one year and five years after aortic repair. Up to 10 years follow-up.
Left: Kaplan-Meier survival estimate.
Right: Forest plot based on HR from appendix 3B Model II, adjusted for all baseline variables and characteristics (gender, age, preoperative hemoglobin and creatinine, bleeding, BMI, smoking, diabetes, hypertension, cerebrovascular, cardiac and respiratory disease).
(DOCX)

S4 Appendix **a**: Survival stratified for transfusion only intraoperatively (blue) or both intra- and postoperatively (Red). Log-Rank test p<0.0001.
**b**: Survival after only receiving transfusion intraoperatively stratified for number of transfusions.
**c**: Survival after receiving transfusion intra- and postoperatively stratified for number of transfusions.
(DOCX)

S5 Appendix Overall 30-day mortality from January 2000 to December 2014, % (N). (DOCX)

S6 Appendix **a-f:** Hazard ratio for postoperative complications by number of transfusions. Note, Figures a (Dialysis) and b (Intestinal ischemia) are depicted using the logarithmic scale.
(DOCX)

S7 Appendix Data set. (XLSX)
