# Supplementary material for: Red blood cell transfusion associated with increased morbidity and mortality in patients undergoing elective open abdominal aortic aneurysm repair
Source: PLoS One. 2019 Jul 11;14(7):e0219263. doi: 10.1371/journal.pone.0219263 (PMC6623955; doi:10.1371/journal.pone.0219263)
Supplement: S1 Appendix — a: Definition of postoperative in-hospital complications. b: Surgical postoperative in-hospital complications. Data are summarized by % (n). (DOCX) [file pone.0219263.s001.docx]

|  | Yes | No |
| --- | --- | --- |
| Dialysis | Postoperative, new onset need for dialysis | No |
| Intestinal ischemia | Intestinal ischemia requiring treatment | No |
| Pulmonary complications | Pulmonary complications requiring treatment | No |
| Cardiac complications | Acute myocardial infarct, new onset arrhythmia, new onset heart failure | No |
| Embolus | Peripheral embolus | No |
| Acute tubulointerstitial nephritis | Postoperative, new onset acute tubulointerstitial nephritis | No |

**Supporting information 1a:** Definition of postoperative in-hospital complications

|  | 0  transfusions | 1 | 2-3 | 4-5 | >5 | All patients |
| --- | --- | --- | --- | --- | --- | --- |
| Dialysis | 0.3 (2) | 0.9 (3) | 0.7 (8) | 1.8 (13) | 8.8 (75) | 2.6 (101) |
| Intestinal ischemia | 0.4 (3) | 0.6 (2) | 1.1 (13) | 2.1 (15) | 7.4 (63) | 2.5 (96) |
| Pulmonary complications | 5.0 (40) | 8.5 (28) | 7.3 (85) | 9.0 (65) | 16.5 (141) | 9.3 (359) |
| Cardiac complications | 3.9 (31) | 4.0 (13) | 6.4 (75) | 6.1 (44) | 15.0 (128) | 7.5 (291) |
| Embolus | 0.6 (5) | 2.1 (7) | 1.6 (18) | 1.1 (8) | 2.8 (24) | 1.6 (62) |
| Acute tubulointerstitial nephritis | 0.8 (6) | 2.4 (8) | 1.4 (16) | 3.5 (25) | 10.3 (88) | 3.7 (143) |

**Supporting information 1b:** Surgical postoperative in-hospital complications. Data are summarized by % (n).
