# Supplementary material for: Red blood cell transfusion associated with increased morbidity and mortality in patients undergoing elective open abdominal aortic aneurysm repair
Source: PLoS One. 2019 Jul 11;14(7):e0219263. doi: 10.1371/journal.pone.0219263 (PMC6623955; doi:10.1371/journal.pone.0219263)
Supplement: S2 Appendix — a: Definition of preoperative comorbidities. The variables were divided in a “Yes” or “No” group as depicted above. Body mass index (BMI) was defined as: BMI = Weight(Kg)(Height(m))2 b: Missing data on preoperative comorbidities % (N). (DOCX) [file pone.0219263.s002.docx]

|  | Yes | No |
| --- | --- | --- |
| Smoking | Currently smoking  Previous smoker | No |
| Diabetes | Diabetes mellitus type 1  Diabetes mellitus type 2 | No |
| Cerebrovascular | Previous transient ischemic attack (TIA).  Amaurosis fugax or stroke. | No |
| Hypertension | Medically well-regulated hypertension. Newly discovered hypertension or poorly regulated hypertension. | No |
| Cardiac | Asymptomatic arrhythmia. Previous acute myocardial infarction. Stable/unstable angina pectoris and/or heart medicine. Uncompensated mb. Cordis. Previous cardiac surgery including percutaneous coronary intervention (PCI) with no symptoms at present. | No |
| Pulmonary | Receiving medical treatment for lung disease.  Dyspnea when speaking or at rest. | No |

**Supporting information 2a:** Definition of preoperative comorbidities

The variables were divided in a “Yes” or “No” group as depicted above.

Body mass index (BMI) was defined as: BMI$=\frac{Weight (Kg)}{{(Height\left( m \right))}^{2}}$

| Missing data, N (%) | 0  N = 801 | 1  N = 329 | 2-3  N = 1 165 | 4-5  N = 724 | >5  N = 857 | All patients  N = 3 876 |
| --- | --- | --- | --- | --- | --- | --- |
| Smoking | 1.4 (11) | 1.5 (5) | 1.4 (16) | 1.4 (10) | 1.2 (10) | 1.3 (52) |
| Diabetes | 0.7 (6) | 0.6 (2) | 0.5 (6) | 0.4 (3) | 0.8 (7) | 0.6 (24) |
| Cerebrovascular disease | 1.4 (11) | 0.6 (2) | 0.9 (10) | 0.8 (6) | 1.5 (13) | 1.1 (42) |
| Hypertension | 1.1 (9) | 0.9 (3) | 1.0 (12) | 0.6 (4) | 1.3 (11) | 1.0 (39) |
| Cardiac disease | 0.8 (6) | 2.1 (7) | 1.2 (14) | 0.7 (5) | 1.5 (13) | 1.2 (45) |
| Pulmonary disease | 0.6 (5) | 0.6 (2) | 0.9 (11) | 0.6 (4) | 0.9 (8) | 0.8 (30) |

**Supporting information 2b:** Missing data on preoperative comorbidities % (N)
