# Supplementary material for: Red blood cell transfusion associated with increased morbidity and mortality in patients undergoing elective open abdominal aortic aneurysm repair
Source: PLoS One. 2019 Jul 11;14(7):e0219263. doi: 10.1371/journal.pone.0219263 (PMC6623955; doi:10.1371/journal.pone.0219263)
Supplement: S3 Appendix — a: Temporal trend in 30-day mortality from January 2000 to December 2014, % (N). During 2000 and 2001 all included patients received a minimum of 1 RBC transfusion. b: Mortality Hazard ratio (95% CI) for dose-dependent subgroups compared to non-transfused patients after excluding patients who did not survive the first 30 days, one year or five years. Model I: Mortality hazard ratio adjusted for age and gender. Model II: Mortality hazard ratio adjusted for all baseline variables and characteristics (gender, age, preoperative hemoglobin and creatinine, bleeding, BMI, smoking, diabetes, hypertension, cerebrovascular, cardiac and respiratory disease). c: Dose-dependent survival for all patients included in the study after excluding the first 30 days, one year and five years after aortic repair. Up to 10 years follow-up. Left: Kaplan-Meier survival estimate. Right: Forest plot based on HR from appendix 3B Model II, adjusted for all baseline variables and characteristics (gender, age, preoperative hemoglobin and creatinine, bleeding, BMI, smoking, diabetes, hypertension, cerebrovascular, cardiac and respiratory disease). (DOCX) [file pone.0219263.s003.docx]

| 30-day mortality | | | |
| --- | --- | --- | --- |
| Year | **No transfusion** | **Transfused patients** | **All patients** |
| 2000 | - | 1.3 (2) | 1.3 (2) |
| 2001 | - | 5.0 (8) | 5.0 (8) |
| 2002 | 0.0 (0) | 4.2 (7) | 3.9 (7) |
| 2003 | 3.6 (2) | 2.6 (5) | 2.8 (7) |
| 2004 | 0.0 (0) | 4.9 (12) | 4.2 (12) |
| 2005 | 0.0 (0) | 3.2 (8) | 2.6 (8) |
| 2006 | 2.0 (1) | 3.0 (8) | 2.8 (9) |
| 2007 | 0.0 (0) | 3.6 (9) | 3.1 (9) |
| 2008 | 0.0 (0) | 3.2 (7) | 2.5 (7) |
| 2009 | 0.0 (0) | 3.9 (10) | 3.4 (10) |
| 2010 | 0.0 (0) | 4.1 (9) | 3.3 (9) |
| 2011 | 2.8 (2) | 5.2 (11) | 4.6 (13) |
| 2012 | 1.1 (1) | 3.6 (6) | 2.7 (7) |
| 2013 | 0.9 (1) | 1.3 (2) | 1.1 (3) |
| 2014 | 1.9 (2) | 4.8 (8) | 3.7 (10) |
| 2000-2014 | 1.1 (9) | 3.6 (112) | 3.1 (121) |

**Supporting information 3a:** Temporal trend in 30-day mortality from January 2000 to December 2014, % (N). During 2000 and 2001 all included patients received a minimum of 1 RBC transfusion.

| Excluding  first: | No transfusion | 1 | 2-3 | 4-5 | >5 |
| --- | --- | --- | --- | --- | --- |
| Model I | | | | | |
| 30 days | 1 | 1.36(1.05-1.75) | 1.26(1.04-1.52) | 1.57(1.30-1.92) | 1.81(1.50-2.20) |
| One year | 1 | 1.29(0.98-1.68) | 1.22(1.00-1.49) | 1.47(1.19-1.81) | 1.60(1.30-1.95) |
| Five years | 1 | 1.43(0.97-2.10) | 1.29(0.96-1.72) | 1.49(1.10-2.02) | 1.59(1.18-2.13) |
| Model II | | | | | |
| 30 days | 1 | 1.22(0.93-1.60) | 1.18(0.96-1.44) | 1.43(1.15-1.78) | 1.42(1.13-1.77) |
| One year | 1 | 1.14(0.86-1.51) | 1.16(0.94-1.42) | 1.37(1.09-1.71) | 1.36(1.07-1.74) |
| Five years | 1 | 1.28(0.86-1.92) | 1.21(0.89-1.63) | 1.37(0.98-1.90) | 1.33(0.94-1.89) |

**Supporting information 3b:**

Mortality Hazard ratio (95 % CI) for dose-dependent subgroups compared to non-transfused patients after excluding patients who did not survive the first 30 days, one year or five years.

**Model I:** Mortality hazard ratio adjusted for age and gender.

**Model II:** Mortality hazard ratio adjusted for all baseline variables and characteristics (gender, age, preoperative hemoglobin and creatinine, bleeding, BMI, smoking, diabetes, hypertension, cerebrovascular, cardiac and respiratory disease).


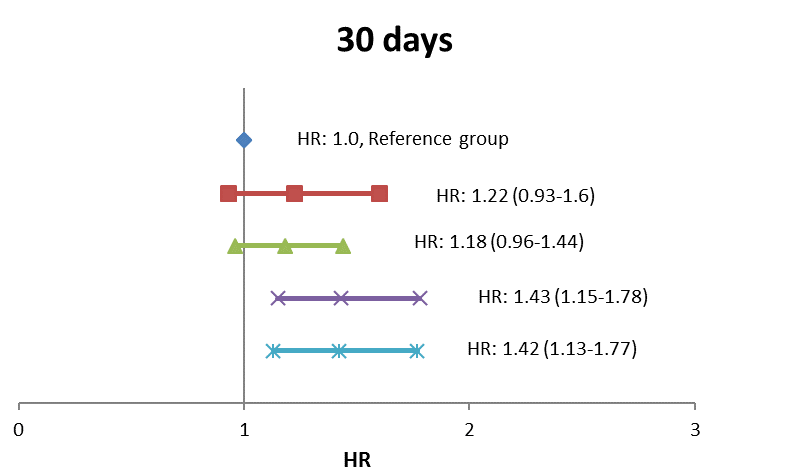

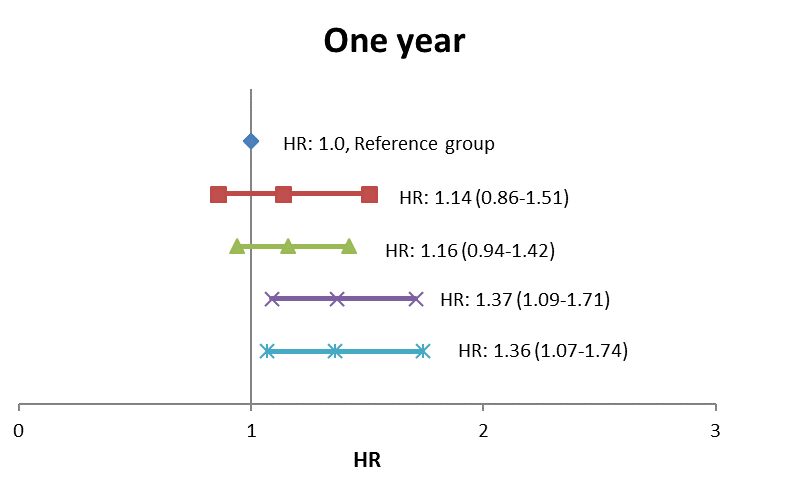

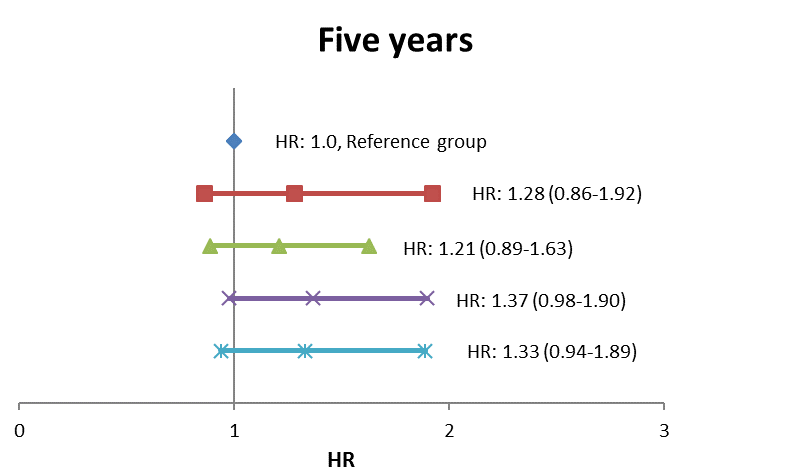


**Supporting information 3c:** Dose-dependent survival for all patients included in the study after excluding the first 30 days, one year and five years after aortic repair. Up to 10 years follow-up.

Left: Kaplan-Meier survival estimate.

Right: Forest plot based on HR from appendix 3B Model II, adjusted for all baseline variables and characteristics (gender, age, preoperative hemoglobin and creatinine, bleeding, BMI, smoking, diabetes, hypertension, cerebrovascular, cardiac and respiratory disease).
