# Supplementary material for: Red blood cell transfusion associated with increased morbidity and mortality in patients undergoing elective open abdominal aortic aneurysm repair
Source: PLoS One. 2019 Jul 11;14(7):e0219263. doi: 10.1371/journal.pone.0219263 (PMC6623955; doi:10.1371/journal.pone.0219263)
Supplement: S4 Appendix — a: Survival stratified for transfusion only intraoperatively (blue) or both intra- and postoperatively (Red). Log-Rank test p<0.0001. b: Survival after only receiving transfusion intraoperatively stratified for number of transfusions. c: Survival after receiving transfusion intra- and postoperatively stratified for number of transfusions. (DOCX) [file pone.0219263.s004.docx]

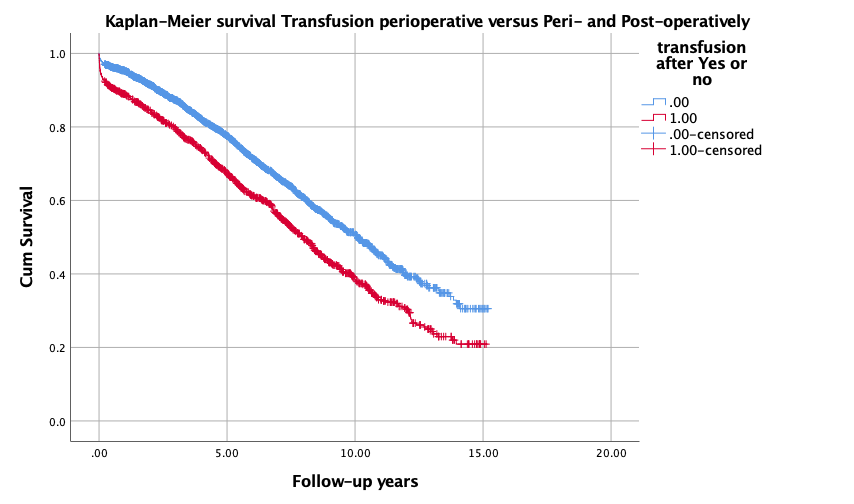

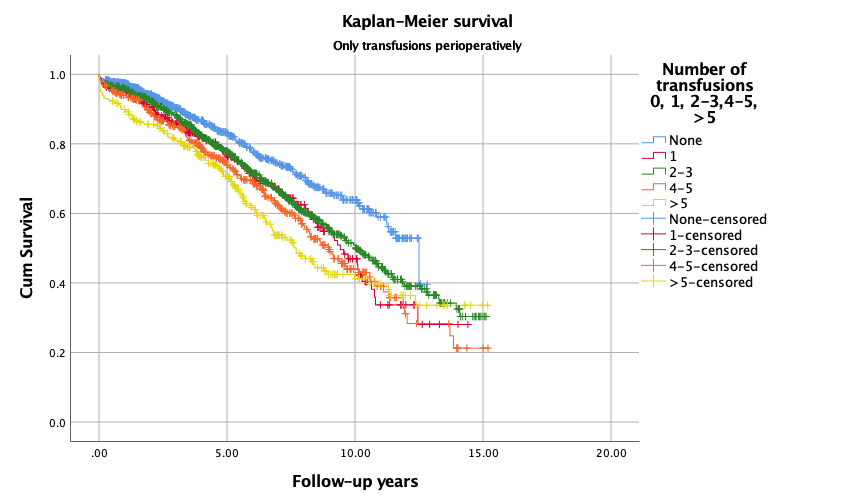

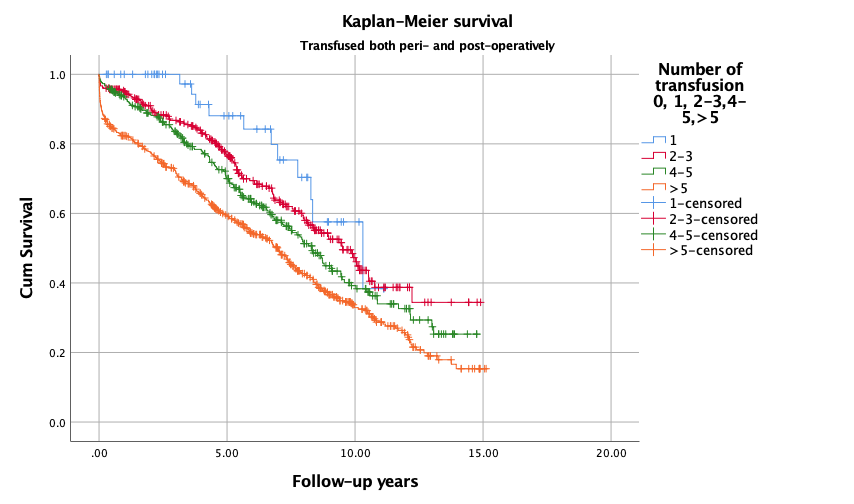


**Supporting information 4, Figure 1**: Survival stratified for transfusion only intraoperatively (blue) or

both intra- and postoperatively (Red)

Log-Rank test p < 0.0001

**Supporting information 4, Figure 2**: Survival after only receiving transfusion intraoperatively

stratified for number of transfusions

**Supporting information 4, Figure 3**: Survival after receiving transfusion intra- and postoperatively stratified for number of transfusions
