# Supplementary material for: Red blood cell transfusion associated with increased morbidity and mortality in patients undergoing elective open abdominal aortic aneurysm repair
Source: PLoS One. 2019 Jul 11;14(7):e0219263. doi: 10.1371/journal.pone.0219263 (PMC6623955; doi:10.1371/journal.pone.0219263)
Supplement: S5 Appendix — (DOCX) [file pone.0219263.s005.docx]

| Number of  transfusions | Number of patients (N) | 30-day mortality  % (N) |
| --- | --- | --- |
| 0 | 801 | 1.1 (9) |
| 1 | 329 | 1.2 (4) |
| 2-3 | 1 165 | 2.2 (26) |
| 4-5 | 724 | 1.9 (14) |
| >5 | 857 | 7.9 (68) |
| All transfused patients | 3 075 | 3.6 (112) |
| All patients | 3 876 | 3.1 (121) |

**Supporting information 5:** Overall 30-day mortality from January 2000 to December 2014, % (N).
