# Supplementary material for: Red blood cell transfusion associated with increased morbidity and mortality in patients undergoing elective open abdominal aortic aneurysm repair
Source: PLoS One. 2019 Jul 11;14(7):e0219263. doi: 10.1371/journal.pone.0219263 (PMC6623955; doi:10.1371/journal.pone.0219263)
Supplement: S6 Appendix — a-f: Hazard ratio for postoperative complications by number of transfusions. Note, Figures a (Dialysis) and b (Intestinal ischemia) are depicted using the logarithmic scale. (DOCX) [file pone.0219263.s006.docx]

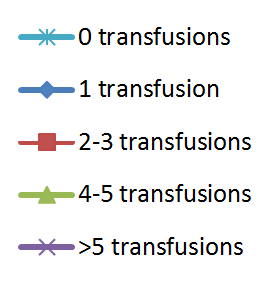


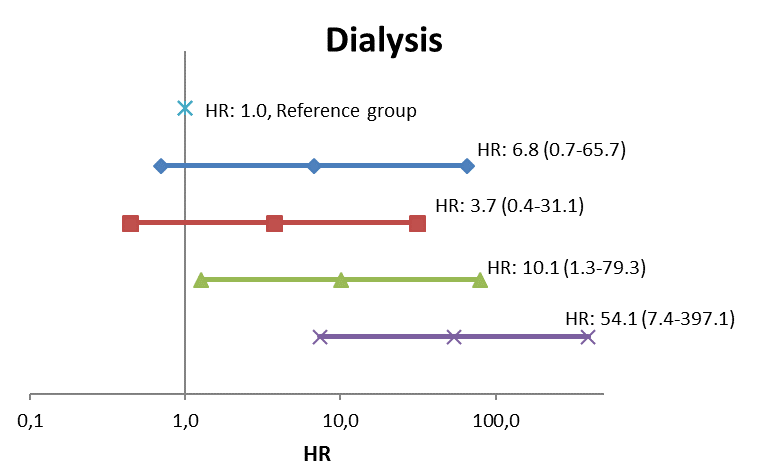

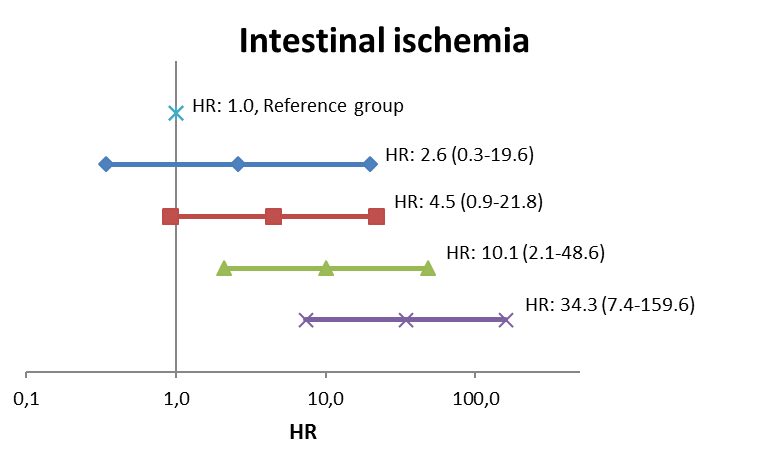

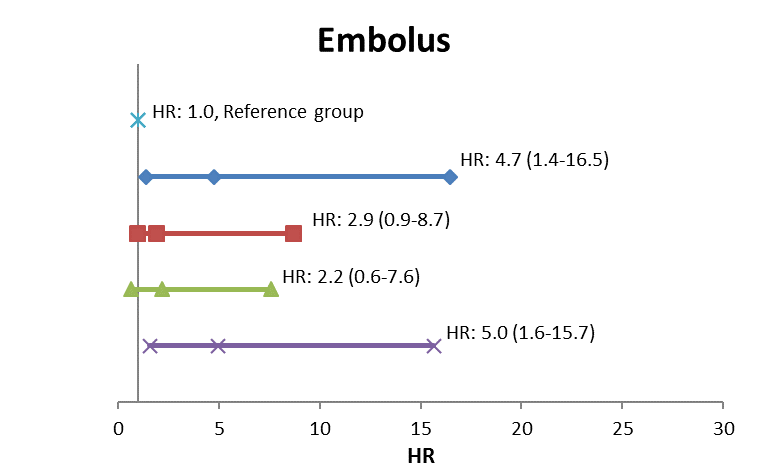

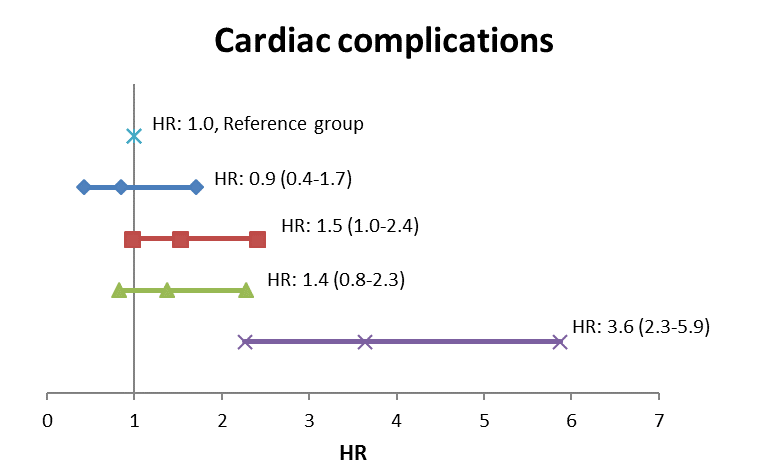

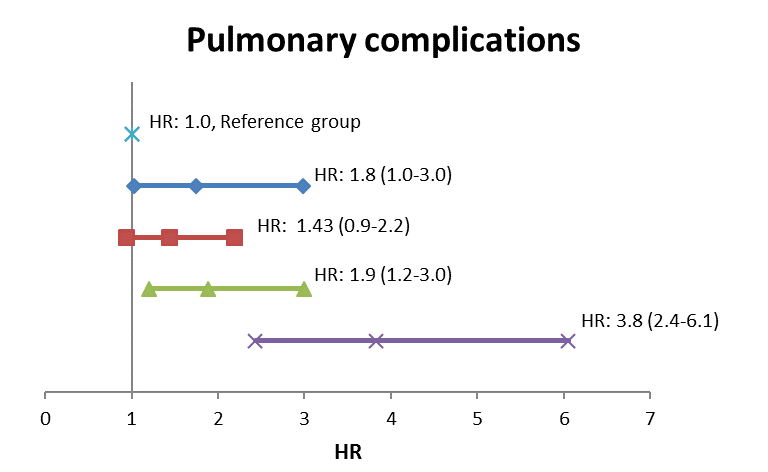

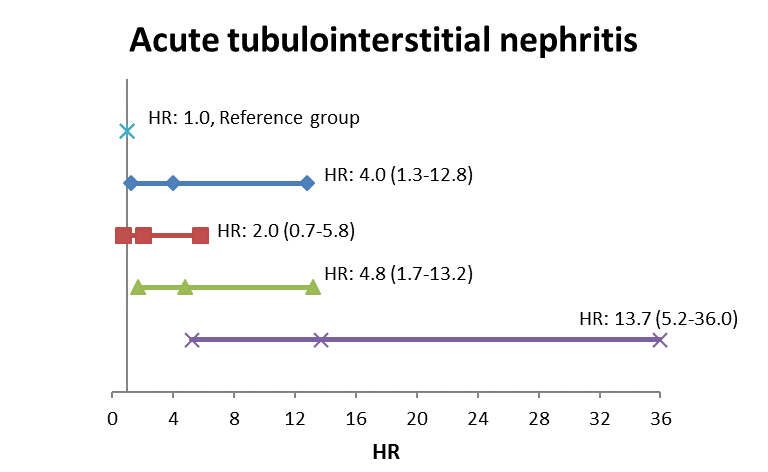


Supporting information 6: Hazard ratio for postoperative complications by number of transfusions. Note, Figures A and B are depicted using the logarithmic scale.
